# The role of critical thinking in explaining students’ academic achievement in undergraduate animal sciences programs

**DOI:** 10.1093/tas/txag119

**Published:** 2026-08-07

**Authors:** Hannah M Twenter, John D Tummons, Jim Spain

**Affiliations:** Division of Animal Sciences, University of Missouri Columbia, Columbia, MO 65211, United States; Division of Applied Social Science, University of Missouri Columbia, Columbia, MO 65211, United States; Division of Animal Sciences, University of Missouri Columbia, Columbia, MO 65211, United States

**Keywords:** critical thinking, undergraduate, GPA, predictive, academic success

## Abstract

Critical thinking skills are an in-demand trait of many STEM professionals, including animal scientists. Many instructors note the importance of critical thinking, but few have a clear understanding of what critical thinking means, how to teach to enhance the development of critical thinking skills, and how to assess the critical thinking as a student learning outcome. Many higher education institutions utilize student success prediction models with the hope that early prediction can be used to provide additional assistance to students when needed to improve the student success rate. These predictive models use high school GPA and standardized test scores, like the ACT and SAT, to predict academic success in college, but models have mixed success in their predictive capacity. The purpose of this study was to determine to what extent differences in critical thinking skills explain a difference between predicted and earned GPAs for first-time animal sciences students. Previous animal sciences students, on average, failed to achieve predicted overall GPAs (*P* < 0.05) and GPAs in STEM courses (*P* < 0.05). Mean student critical thinking scores of first-time animal sciences students were significantly (*P* < 0.05) below normative data. Current animal sciences students significantly (*P* < 0.05) underperformed their predicted GPA after one semester, but differences between predicted and earned GPA after four semesters were not significant (*P* > 0.05). The current university-provided predicted GPA model using standardized tests and high school GPA was significant *F = *68.13(1,103, *P* < 0.001) and accounted for 39% of the variance (*adjusted R^2^* = 0.392) for rising junior students. The second model included students’ critical thinking, graduating class size, and involvement in youth agriculture clubs. Model 2 was also significant *F = *17.82(3,100, *P* < 0.001, adjusted *R^2^* = 0.393). No added covariates in Model 2 were significant (*P* > 0.05) and the additional factors did not improve the predictive ability of the model (Δ*F = *1.028(3,100), *P* > 0.05*)*. Researchers recommend future research exploring the developmental factors of youth critical thinking, why predictive models overestimate GPA, grading policies, and interpersonal variables which can account for the unexplained variance in the prediction model.

## Teaser

The ACT and high school GPA of animal sciences undergraduate students predicts 39% of the variance in their undergraduate GPA. Students lack critical thinking skills, but critical thinking skills were not predictive of student GPAs.

## Introduction

Critical thinking is a responsibility all citizens have in determining valuable and true information to help navigate their everyday lives (Ten Dam and Volman 2004; Butler and Halpern 2020). Critical thinking involves purposeful, self-regulatory judgment–the cognitive process that uses analysis, interpretation, explanation, inference, evaluation, and reflective self-correction, by which people–individually or thinking and working together–decide what to believe or what to do (Facione 2011). A lack of critical thinking is a salient issue, as many citizens have developed an increasing distrust in science which threatens scientific advancement and policy (Tsipursky et al. 2018). Many in higher education feel critical thinking is a practical skill students need to achieve academic success (Diamond 1997; Pithers and Soden 2000; AACU 2010). The goal of higher education is to help graduates perform critical analyses and make independent, real-world decisions (Moore 2004; Barrie 2006; Karantzas et al. 2013). Over 93% of faculty in the U.S. identified critical thinking as the most important skill in undergraduate education (Pithers and Soden 2000; Bok 2006). However, graduates entering the work force often lack the ability to think critically to solve complex, multidisciplinary, real-world problems (Candy and Crebert 1991; Schmidt 1999; Dunne and Rawlins 2000; Behar-Horenstein and Niu 2011).

Critical thinking skills are an in-demand trait of those in STEM disciplines, including animal sciences (Duron et al. 2006; Oliver et al. 2010; Flores et al. 2012; Prinsley and Baranyai 2015). Critical thinking skills can change over time, with measurable critical thinking development occurring in as little as one semester (Lundy et al. 2002). Animal sciences students can gain critical thinking skills as they progress through their coursework (Smith et al. 2020). Researchers have identified several demographics which may influence students’ critical thinking skills, including age, gender, their GPA, involvement in on-campus clubs, and interaction with faculty and peers (Gellin 2003; Ricketts and Rudd 2005; White et al. 2012).

Many instructors note the importance of critical thinking, but few have an idea of exactly what it is, how it should be taught, or how to assess it (Paul et al. 1997). Key critical thinking skills include interpretation, analysis, inference, evaluation, explanation, and self-regulation (Facione 2011). Critical thinking skills are not something the students simply acquire through exposure to classroom content in lecture (Glaser 1941; Anderson 1942; Taba 1962, Arnold et al. 2018; Nickerson 1989; Newman 1990). Students’ critical thinking skills can develop when instructional techniques allow students to begin to think critically in applying and analyzing content (Feuerstein 1980, Lundy et al. 2002, Stedman and Adams 2012; Doyle 1983). Teachers can foster critical thinking development by asking students to answer questions following in-class presentations and employing case-based teaching strategies (Ferree et al. 2022; Norris-Parish et al. 2024).

College GPA is a ubiquitous measure of college success and career readiness. Critical thinking skills are positively associated with college GPA, and GPA can serve as a significant predictor of critical thinking ability in students (Giancarlo 1996; Jenkins 1998; Ricketts and Rudd 2005). For college students, perceived academic control was predictive of critical thinking dispositions and GPA, although academic control was a more accurate predictor than critical thinking for college GPA when controlling for high school GPA (Stupnisky et al. 2008). Many higher education institutions utilize GPA predictive models based on standardized testing scores and high school GPA for new and prospective students to improve student success rates and to assign advising groups (Tierney et al. 2009; Allensworth and Clark 2020; Alyahyah and Düştegör 2020). However, these models do not fully explain academic outcomes; standardized tests, such as the SAT and ACT, are not perfectly correlated with college GPA. Multiple studies show high school GPA is a stronger predictor of college readiness than standardized test scores (Geiser and Santelices 2007; Kobrin et al. 2008; Bowen et al. 2009; Hiss and Franks 2014). Some differences may be attributed to personal factors beyond ACT and GPA. Students in small, rural schools and schools with high minority populations have less access to STEM classes and experience 59% lower success rates on AP tests (Grant et al. 2025). In some instances, college success (as measured by GPA) is perhaps more correlated with a students’ socio-economic background than academic ability, indicating a need for more comprehensive ways to predict college success (Rothstein 2004; Geiser and Santelices 2007).

The experiential learning provided in agricultural education programs like FFA and 4-H help students develop critical thinking skills (Rayfield et al. 2012) and positively influence students’ educational outcomes including their academic achievement, skill attainment, and college retention rates (Dyer et al. 1996; Dyer and Breja 1999). Ball et al. (2001) also noted students involved in these programs typically achieve higher scores on standardized tests, like the ACT, and had stronger college cumulative GPAs. Students who are actively involved in extra-curricular activities, like these programs, are less likely to underachieve, and their participation in such activities enhances their critical thinking skills (Reis 1998; Reis and McCoach 2000; Gellin 2003; Shann et al. 2006), suggesting youth agriculture program involvement could provide additional insight into a predictive model. The purpose of this study is to determine to what extent critical thinking skills can explain differences between predicted and earned GPAs for first-time college students in an animal sciences program at a Midwestern land-grant university. In this study, a first-time college student is defined as a recent high-school student who is attending a college or university for the first time but notes they may also have already earned college credit through dual credit or earned credit options, such as advanced placement test credit (2026).

## Research objectives

1. Describe critical thinking skills (overall critical thinking skills, analysis, interpretation, inference, evaluation, explanation, induction, deduction, numeracy) of first-time college students majoring in animal sciences during their first semester.
2. Describe the STEM GPA of first-time college students majoring in animal sciences during their first semester.
3. Describe the overall GPA of first-time college students majoring in animal sciences during their first semester.
4. Calculate the historical variation between overall predicted GPA and earned GPA of animal sciences first-time college students for students from 2014 to 2019.

*H_01_*: There was not a significant difference (*P* > 0.05) between the historical overall predicted GPA and the earned GPA of first-time college students majoring in animal sciences from 2014–2019.

5) Calculate the variation between overall predicted GPA and earned GPA of first-time college students majoring in animal sciences during their first semester entering Fall 2023.

*H_02_*: There was not a significant difference (*P* > 0.05) between the overall predicted GPA and the earned GPA of animal sciences first-time college students in Fall 2023.

6) Compare animal sciences first-time college students’ California Critical Thinking Skills Test (CCTST) scores to the nationwide normative data.

*H_03_*: There was not a significant difference (*P* > 0.05) between first-time college students majoring in animal sciences’ CCTST scores to the nationwide normative data.

7) Determine to what extent differences in critical thinking scores, youth involvement, and high school class size explain differences between overall predicted GPA and earned GPA of first-time college students majoring in animal sciences.

*H_04_*: Differences in critical thinking scores, youth involvement, and high school class size did not explain a significant difference (*P* > 0.05) between overall predicted GPA and earned GPA of first-time college students majoring in animal sciences.

8) Compare the critical thinking scores of students who were retained in the major, those who switched majors, and those who were not retained at the university.

*H_05_*: No significant difference (*P* > 0.05) between critical thinking scores of first-time college students majoring in animal sciences retained in the major, those switching majors, and those not retained at the university.

## Materials and methods

### Participation and inclusion

Prior to contacting students, all documentation and procedures were approved by the Institutional Review Board (IRB) project #2091055. Students provided active consent prior to participating in any observations or examinations. The target population for this study was first-time college students majoring in animal sciences at a Midwest land-grant institution. The accessible population consists of all first-time college animal sciences students beginning in the fall of 2023 (*N* = 135). Researchers also sourced baseline GPA data from students in the animal sciences program enrolled as first-time college students over the past decade (*N* = 690) using data from the institution’s student information system (SIS). Historical dates were intentionally chosen to stop before the COVID era. Prior GPA and advising group data were collected from the student information system to determine academic achievement and predictive capability of the advising group model currently used for the university. Students were contacted both in-person during their first animal sciences course and via e-mail requesting their participation in the study.

### Implementation of CCTST

**Figure 1:**
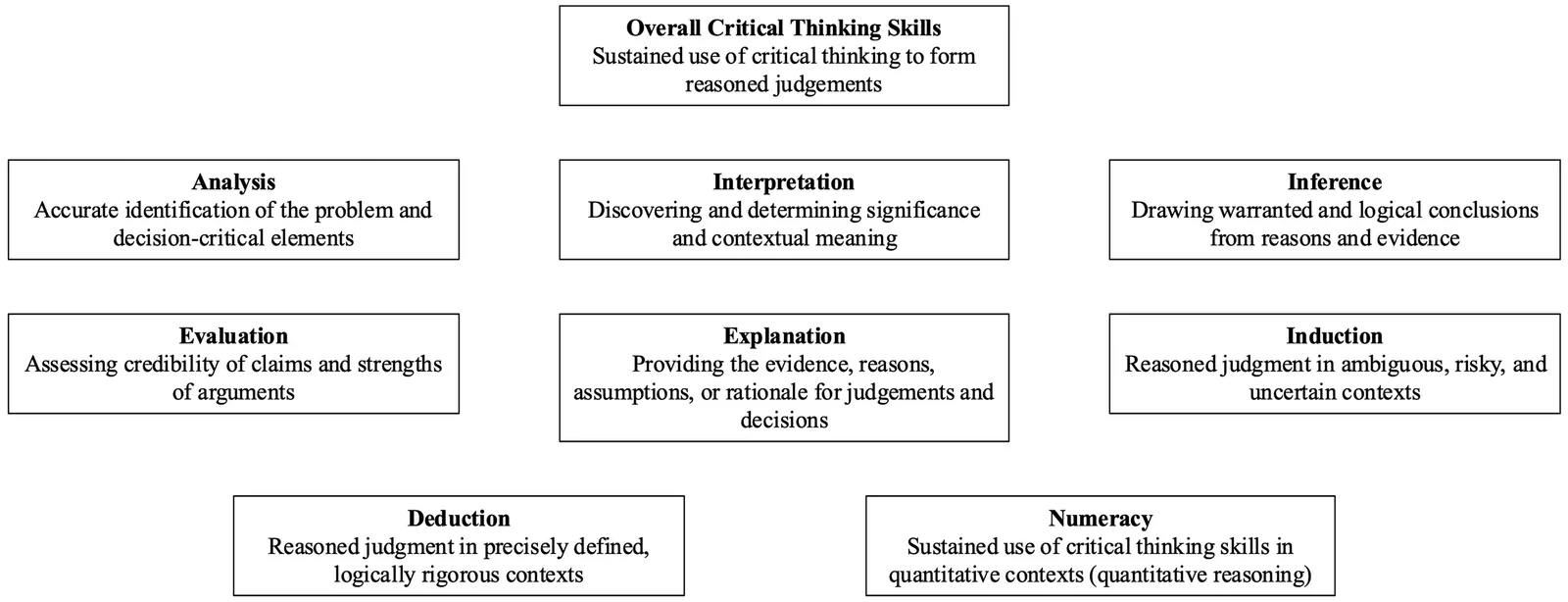
The components of critical thinking skills assessed during the California critical thinking skills test and their role in a student’s earned GPA (Insight Assessment).

Researchers utilized the California Critical Thinking Skills Test (CCTST 2023), hosted on an online platform by Insight Assessment, to estimate critical thinking across eight dimensions. The CCTST uses 40 scenario-based questions to determine a student’s critical thinking ability based on a 100-point scale, generating an overall critical thinking score and eight sub scores (see Fig. 1). The CCTST skills are qualitatively described in scale anchors as *not manifested* (50 to 62), *weak* (63 to 69), *moderate* (70 to 78), *strong* (79 to 85), and *superior* (86 to 100). Insight Assessment provided reliability data; this instrument exceeds the minimum 0.70 alpha reliability estimate coefficient threshold suggested by Nunnaly (1978).

We also collected participant demographic data, including their involvement in agricultural youth organizations (FFA, 4-H), graduating class size, and consent for researchers to access their GPA information. We also requested student financial need (high, medium, low, none, as defined by the Office of Financial Aid), first generation college (yes/no, one whose parents do not have a degree from a college or university), and previous coursework (yes/no, if students transferred any credit into the university prior to enrollment).

Additionally, researchers collected their STEM GPA taken at the university (dual credit courses excluded), overall GPA, and predicted GPA using the student information system (SIS) at the study’s institution. The predicted GPA at this university is intended to predict GPA for the first year of enrollment. All information collected remained confidential and was provided with written consent from students and official parties at the study institution.

### Data collection

Students enrolled as first-time college students majoring in animal sciences begin the curriculum with an introductory course in animal sciences their first semester at the study institution. The Fall 2023 students were asked to complete the CCTST during their first laboratory session of the semester using their own computer and to avoid using their phones or any other sources to complete questions in the assessment tool. Participants provided written consent when answering the identifier questions, and those who answered no were removed from the study. Students who did not attend the first semester’s laboratory session were contacted the second week of the semester and invited to participate, but none of those students responded. An overall response rate of 87% (*n* = 118) was achieved.

All Fall 2023 student participants provided their full name when completing the CCTST for data organization. All information from the identifier questions was provided directly from the participants to the investigator from Insight Assessment. All scales related to the CCTST were converted to a numerical value and provided upon request from Insight Assessment. Data from the institution’s student information system for the Fall 2014 to 2019 cohort required a request form from the study institution’s administrative faculty, and once filed, all requested data were provided via email to the investigator.

### Data processing

Data were analyzed using SPSS. A paired samples *t*-test was utilized to compare the historical variation in predicted GPA to earned GPA of first-time animal sciences students prior to the spring of 2020. For Fall 2023 students, we compared predicted GPA scores to earned GPA after one semester and four semesters. Critical thinking scores were compared against a national normative critical thinking score of college students. The researcher established a national average by running a linear regression of the percentile ranking of overall scores provided by CCTST to determine a score for the 50th percentile. A stepwise multiple regression was used to compare factors between the student participants and determine if critical thinking can be a predictor of success. Effect size was evaluated using the adjusted *R*^2^ where applicable. Level of significance was set a priori at *P* < 0.05.

## Results

The author collected demographic information from the accessible population to better describe participants. The 118 participating first-time college students enrolled in the fall of 2023 consisted of more females (*f = *99) than males (*f = *19). Twenty-six (22.03%) of the students were first-generation students (see Table 1).

**Table 1:** Demographic characteristics of first time college students starting Fall 2023 (*n* = 118).

		*f*		%
**Sex**				
Male		19		16.10%
Female		99		83.90%
**First-Generation**				
Yes		26		22.03%
No		92		77.97%
**Financial Need**				
High		38		32.20%
High/Medium		10		8.47%
Medium/Low		7		5.93%
Low/No		53		44.92%
Unknown		10		8.47%
**Previous College Coursework**				
Yes		97		82.20%
No		21		17.80%

Previous college coursework would apply to dual credit and advanced placement credit earned while in high school.

Of the 690 historical participants, majority of them were also female (*f = *567) rather than male (*f = *123). This cohort of students consisted of 227 (*N* = 32.90%) first-generation students. Student participants were varied in their financial need with the status of some students unknown (see Table 2).

**Table 2:** Demographic characteristics of first time college students FS2014—FS2019 (*n* = 690).

		*N*	%
**Sex**			
Male		123	17.83%
Female		567	82.17%
**First-generation**			
Yes		227	32.90%
No		463	67.10%
**Financial need**			
High		245	35.51%
High/Medium		88	12.75%
Medium/Low		42	6.09%
Low/No		244	35.36%
Unknown		71	10.29%
**Previous college coursework**			
Yes		215	31.16%
No		475	68.84%

Previous college coursework would apply to dual credit and advanced placement credit earned while in high school.

Research objective one sought to describe critical thinking skills (overall critical thinking skills, analysis, interpretation, inference, evaluation, explanation, induction, deduction, numeracy) of animal sciences first-time college students. For each component of the CCTST, the author calculated the mean score as well as median, mode, variance, and range. The overall critical thinking skills score (*µ* = 72.14, *SD = *5.85) indicates a sustained use of critical thinking when forming reasoned judgements (see Table 3). The test also generates a score of eight cognitive skills associated with critical thinking. Of those various constructs, students achieved a *moderate* score for induction skills. Induction refers to reasoned judgement in ambiguous, risky, or uncertain contexts (*µ* = 75.25, *SD = *6.41). Students, on average, also earned *moderate* scores for interpretation. Interpretation is defined as the student’s ability to discover and determine both significant and contextual meaning (*µ* = 75.05, *SD = *7.46). Other *moderate* skills include explanation, inference, analysis, and evaluation. Explanation infers an ability to provide evidence, reason, assumption, or rationale for their judgements and decisions (*µ* = 73.23, *SD = *8.27). Inference is their ability to draw warranted and logical conclusions from reason and the evidence (*µ* = 72.39, *SD = *6.44). The analysis portion of the test indicates a student’s ability to accurately identify the problem and other decision-critical elements (*µ* = 72.12, *SD = *7.79). The evaluation score indicates a student’s ability to assess the credibility and claims and the strength of the argument (*µ* = 71.77, *SD = *7.05). Students earned a *weak* score in deduction and numeracy. Deduction refers to reasoned judgement in precisely defined and logically rigorous contexts (*µ* = 69.99, *SD = *5.74). Numeracy (quantitative reasoning) indicates a student’s sustained use of critical thinking skills in quantitative contexts (*µ* = 69.42, *SD = *7.01).

**Table 3:** Descriptive statistics of CCTST scores^a^ from first-time college students starting Fall 2023 (*n* = 118).

			*µ*	*SD*	Median	Mode	Range
**Construct Score**							
Overall			72.14	5.85	72	68	59–89
Induction			75.25	6.41	75	70	60–93
Interpretation			75.05	7.46	75	80	59–92
Explanation			73.23	8.27	71	75	55–96
Inference			72.39	6.44	73	73	55–91
Analysis			72.12	7.79	71	67	55–96
Evaluation			71.77	7.05	71	75	59–92
Deduction			69.99	5.74	69	69	56–90
Numeracy			69.42	7.01	69	65	55–90

^a^The CCTST skills are qualitatively anchored as *not manifested* (50–62), *weak* (63–69), *moderate* (70–78), *strong* (79–85), and *superior* (86–100).

Research objective two sought to describe the first semester STEM GPA of animal sciences first-time college students. This objective was answered from the perspective of first-time college students enrolled as an animal sciences major at a Midwestern land-grant institution beginning in the fall of 2023 (*n* = 118). The students had a mean STEM GPA of 2.99 (*SD = *0.91) on a scale of 0–4, achieving just below a “B” letter grade in their first-semester STEM courses. The GPAs ranged from 0.00 to 4.00. The median STEM GPA was 3.205, indicating most students performed at a “B” letter grade, and the mode STEM GPA was a 4.00, suggesting the GPAs may be skewed toward higher grades rather than lower grades (see Table 4).

**Table 4:** Academic characteristics of animal sciences first-time college students FS2023 (*N* = 118).

		*µ* (*SD)*	Median	Mode	Range
**GPA**					
STEM		2.99 (0.91)	3.205	4.0	0.0–4.0
Overall		3.10 (0.85)	3.35	4.0	0.0–4.0

Research objective three sought to evaluate the students’ overall GPA. This metric included all courses, including STEM courses. Students achieved an overall first-semester GPA higher (*µ* = 3.10) than that of their STEM-specific GPA with an average letter grade just above a “B”. The GPAs ranged from 0.00 to 4.00. The distribution of these grades mirror that of the STEM GPAs in that the median and mode were higher than the mean (*Med = *3.35, *Mo = *4.00).

Research objective four used historical data from first-time college students enrolled in animal sciences beginning in the fall semesters of 2014 through 2019 (*N* = 690) to evaluate the predictive capability of the current model. A comparison of the students’ predicted GPAs and earned GPAs was conducted using a paired-samples *t*-test. The mean predicted GPA for these students was 3.14 (*SD = *0.41), but the earned GPA was 2.95 (*SD = *0.85). The results of the *t*-test indicated a lower (*P* < 0.0001) earned GPA than what was predicted (*t*(689) = 7.75); therefore, the author rejected null hypothesis one. The author used a Shapiro-Wilks test to assess the normality of the dataset. The results indicated the data were not normally distributed (*W = *0.93, *P* < 0.0001); however, according to the central limit theorem, a sample size of 100 or more observations mitigates the concerns about the violation of normality (Altman and Bland 1995; Ghasemi and Zahediasl 2012).

To determine the variation between the predicted GPA and earned GPA of animal sciences first-time college students enrolled in the fall of 2023, the author used two paired-samples *t*-test. For the first test, the predicted GPA was compared to the earned first-semester GPA (*n* = 118). This cohort of students had an average predicted GPA of 3.30 (*SD = *0.34) and earned an average overall first-semester GPA of 3.10 (*SD = *0.85). This cohort of students had a significant (*P* < 0.05) difference between their predicted and earned GPAs and did not perform as well as anticipated (*t*(117) = 3.05). The author rejected null hypothesis two, stating there was no significant difference between predicted and earned GPA.

Researchers revisited these data after four semesters of student coursework to determine how GPAs had changed for those students still enrolled at the University (*n* = 105). This cohort of students had an average predicted GPA of 3.36 (*SD = *0.32) and earned an average overall GPA of 3.32 (*SD = *0.85). Researchers failed to reject the null hypothesis (*t*(104) = 0.803, *P* > 0.05), stating significant differences exist between predicted GPA and earned GPA for students after four semesters of coursework. For students who were no longer enrolled at the university (*n* = 13), the predicted GPA was significantly lower (*x̄* = 2.88, *P* < 0.05) than those retained. To assess the normality of the dataset, the author used a Shapiro-Wilks test. The results indicated the data were not normally distributed (*W = *0.90, *P* < 0.0001); however, the central limit theorem states that if a sample size has 100 or more observations, a violation of normality is of less concern (Altman and Bland 1995; Ghasemi and Zahediasl 2012).

**Figure 2:**
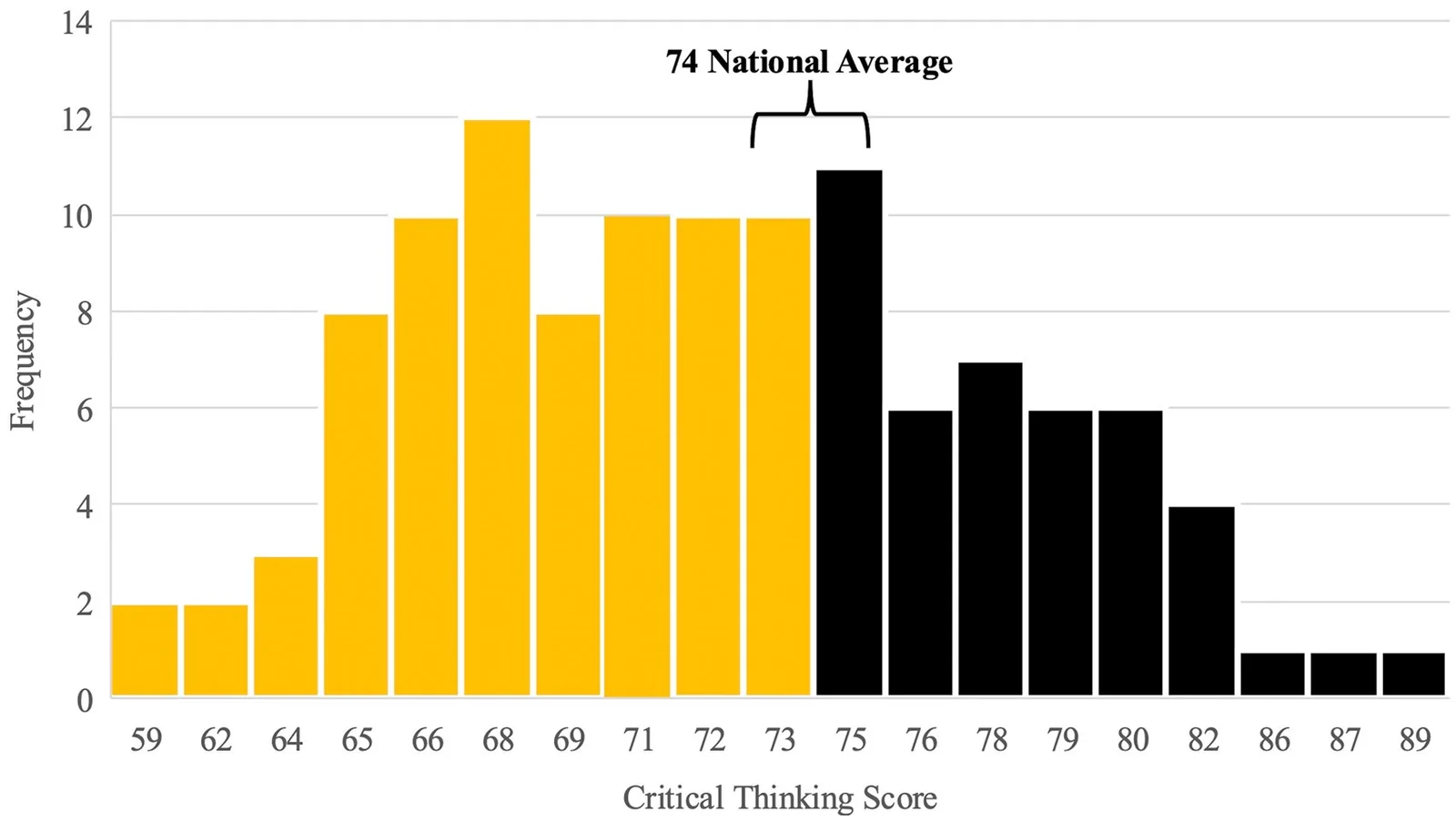
Distribution of overall critical thinking scores among animal sciences first-time college students enrolled fall 2026 (*n* = 118).

Research objective six sought to compare the Fall 2023 animal sciences first-time college students’ CCTST scores to the nationwide normative data provided by the instrument publisher. To accomplish this, the researcher employed a one-sample *t*-test for statistical analysis. The national average, standard deviation, and standard error were not provided by the Insight Assessment. The first-time college students from the Midwest land-grant institution had an overall critical thinking score below (*P* < 0.05) the national average (*t*(117) = −3.31). Most students (66.56%) did not meet the national average score (Fig. 2).

For objective seven, the author identified critical thinking skills, class size, and youth agricultural club involvement as independent variables of interest and overall GPA as the dependent variable. A hierarchal multivariate regression was used to determine what differences in overall GPA are explained by differences in critical thinking, class size, and youth agricultural dependent club involvement when controlling for predicted GPA (core high school GPA and standardized test score (ACT/SAT)).

Field (2009) identified nine assumptions which must be true to draw conclusions about a population using regression modeling. The first assumption for regression analysis is variable type: all predictor variables must be categorical or interval data with no constraints on the variable’s outcome. The investigator verified that critical thinking skills, class size, agricultural youth club involvement, and predicted GPA were all either categorical or approached interval data; therefore, assumption one was assumed to be met.

Assumption two required for the regression is the predictors should have some variation in value. The investigator identified one potential covariate (first-time college student) which did not vary among participants; thus, all non-first-time college students were not included in the study. All other independent variables varied among participants. Therefore, the author concluded assumption two was tenable. Assumption three required for reporting regression analysis is the assumption of no perfect multicollinearity. To test the assumption of multicollinearity, the author reviewed the VIF (variance inflation factor) and tolerance statistics generated in SPSS for the regression model entered (Field 2009). The investigator found no perfect multicollinearity, as VIF values were less than 10, and all tolerance statistics exceed the 0.20 threshold (Field 2009). The researcher accepted the assumption of no perfect multicollinearity as tenable (see Table 5). Because the average VIF statistic exceeded 1.0, the researcher acknowledged that multicollinearity may influence the regression equation (Bowerman and O’Connell 1990).

**Table 5:** Tolerance and VIF collinearity statistics for multivariate regression for overall GPA (*n* = 105).

	PGP	Critical thinking score	Class size	Youth Club Involvement^a^
**Overall GPA**	0.73 (1.36)	0.79 (1.28)	0.88 (1.14)	0.91 (1.10)

^a^Youth Club Involvement was coded *as 1 = participated in agricultural youth clubs* and *2 = did not participate in agricultural youth clubs*.

Assumption four requires that predictors are uncorrelated with ‘external variables’, or variables which have not been included within the regression model which influence the dependent variable. The inquirer addressed assumption four by reviewing academic success research and accounting for predicted GPA by entering predicted GPA as a covariate in the first step of the hierarchal regression model. The author concluded assumption four was tenable. Assumption five assumes homoscedasticity, or constant variance of the residuals at each predictor level. The assumption of homoscedasticity was tested by examining the residuals for interval variables (Field 2009). The author also reviewed the normal probability plot of the residuals for the interval predictor variables and determined assumption five was tenable (Field 2009). Assumption six for regression analysis is the independence of observations. This assumption was evaluated using the Durbin-Watson test. The Durbin-Watson statistic ranges from 0 to 4, with a value close to 2 indicating no autocorrelation (Durbin and Watson 1950). The test yielded a value of 2.032, which falls within the acceptable range of 1 to 3 for the dependent variable of actual GPA earned as a rising junior; therefore, the researcher assumed assumption six was tenable. Assumption seven for regression analysis is normal distribution of error. The normal distribution of error assumes model residuals are random, normally distributed variables with a mean of zero. The author tested normal distribution using histograms and P-P plots and visually confirmed the normality of residual data (Field 2009). The assumption of normal distribution of error was tenable and assumption seven was met. Assumption number eight for regression analysis is independence of all values of the outcome variable. With only one outcome variable, the author assumed the outcome variable independence requirement was met. Assumption nine was linearity of the outcome variable. To test for linearity, the author plotted the standardized residuals against the standardized predicted values. The author visually confirmed the data appeared as a random array of dots and not a waved-shaped line. The author accepted the assumption of linearity as tenable for this study.

To test null hypothesis four, the researcher performed a hierarchical multiple regression analysis with the constructs of predicted GPA, overall critical thinking score, graduating class size, and involvement in youth agriculture clubs as independent variables and overall GPA following four semesters of classes as the dependent variable. The author created two models; the first of which utilized the current predictor model at the study institution and included the students’ predicted GPAs. The second step created a model that simultaneously included students’ overall critical thinking scores, graduating class size, and involvement in youth agriculture clubs.

In Model 1, the investigator regressed students’ predicted GPA against the earned GPA of students retained at the university following two years of coursework (rising juniors *n* = 105, see Table 6). The model was significant, *F = *68.13(1,103, *P* < 0.001) and the predicted GPA explained 39.2% of the variance in GPA after four semesters of coursework (adjusted *R^2^* = 0.392).

**Table 6:** Hierarchical regression of overall junior GPA differences explained by critical thinking, class size, and youth agricultural club involvement (model 2), when predicting for predicted GPA (model 1) (*n* = 105).

	Model 1			Model 2		
Variable	*β*	*t*	*P*	*β*	*t*	*P*
**(Constant)**		−0.624	0.534		−1.456	0.149
**PGP**	0.631	8.254	0.001^*^	0.571	6.381	0.001^*^
**Critical thinking score**				0.135	1.568	0.120
**Class size**				0.020	0.245	0.807
**Youth Club Involvement^a^**				0.061	0.765	0.446
***R***	0.631			0.645		
***Adjusted R^2^***	0.392			0.393		
***R^2^ change***				0.026		
***F change***	68.129^*^(1,103)			1.028(3,100)		

^a^Youth Club Involvement was coded as *1 = participated in agricultural youth clubs* and *2 = did not participate in agricultural youth clubs*.

* = *P* < 0.05.

The second model included students’ critical thinking, graduating class size, and involvement in youth agriculture clubs. Model 2 was also significant *F = *17.82(3,100, *P* < 0.001) and slightly increased the predicted variance in first-semester GPA performance (adjusted *R^2^* = 0.393). No additional predictor variables in Model 2 were significant (*P* > 0.05).

Null hypothesis four stated differences in critical thinking scores did not explain a significant difference between overall predicted GPA and earned GPA of animal sciences first-time college students. The researcher tested null hypothesis four by examining the significance of change in F between Model 1 and 2. The author identified a non-significant change in *F = *1.028 (3,100, *P* = 0.384) generated between Model 1 and Model 2; therefore, researchers failed to reject the null hypothesis. The adjusted *R^2^* accounted for about 39.2% of the variance in Model 1. In Model 2, the adjusted *R^2^* accounted for about 39.3% of the variance. There was not a significant difference between the two models.

Of the 118 students who began the study, 89 students continued their studies in animal sciences after four semesters, while 16 students remained at the University in a different major, and 13 students no longer attended the university. Researchers calculated predicted GPA for retained students ( *x̄* = 3.36, *SD = *0.30), those who switched majors (*x̄* = 3.29, *SD = *0.31), and those who were no longer students at university (*x̄* = 2.88, *SD = *0.36) (see Table 7). Actual GPA for retained students was 3.32 (*SD = *0.49), whereas those who switched majors was 3.25 (*SD = *0.31). Critical thinking scores for the three groups were 72.71 (*SD = *5.93) for retained students, 70.06 (*SD = *5.51) for those who switched majors, and 70.85 (*SD = *5.26) for those who were no longer students. Researchers used ANOVA to compare means of the three groups. Researchers failed to reject the null hypothesis, as no significant (*P* > 0.05) differences were found between the three groups of students (*F*(2,117) = 1.772, *P* > 0.05, *η*^2^ = 0.03).

**Table 7:** Comparison of academic characteristics among retained animal sciences students (*n* = 89), switching majors (*n* = 16), and no longer enrolled (*n* = 13).

		Predicted GPA		Actual GPA		Critical thinking	
		*x̄*	*SD*	*x̄*	*SD*	*x̄*	*SD*
**Retained students**		3.36	(0.30)	3.32	(0.49)	72.71	(5.93)
**Switch majors**		3.29	(0.31)	3.25	(0.31)	70.06	(5.51)
**No longer students**		2.88	(0.36)	N/A		70.85	(5.26)

## Conclusions/implications

This study utilized a purposive sample of two cohorts of students at a Midwestern land-grant university. The researcher identified participants as a representative time and place sample of the population justifying the use of inferential statistics for data analysis (Oliver and Hinkle 1982). Linder et al. (2001) concluded when response rates are ≥ 85%, no control for nonresponse error is needed. This study had an 87% response rate; thus, these findings may be inferred with caution to future cohorts of freshman animal sciences students.

First-time college students majoring in animal sciences exhibited at the start of their first semester, on average, *moderate* critical thinking skills. We conclude freshman students significantly underperformed in critical thinking when compared to normative data from peers of various academic levels in four-year institutions. Specifically, students were *weak* in deduction and numeracy. As indicated by range data, we conclude at least one student was *not manifested* in every construct, and overall. Animal sciences coursework requires students to work with a variety of quantitative measurements, then apply this knowledge. Given students were *weak* in deduction and numeracy, they may struggle to employ reasoned judgement and sustained use of critical thinking skills in a quantitative context. Considering over half of the first-time college student participants enrolled in the fall 2023 semester did not meet normative critical thinking scores, an implication for instructors is to assume not all students enter the program with moderate levels of critical thinking. Critical thinking is a skill that can develop over time and has been documented with undergraduate animal science students (Lundy et al. 2002; Smith et al. 2020). Instructional adjustments may include remediation, intentional coaching, and supporting instruction in critical thinking (Halpern 2007).

Researchers also sought to measure the accuracy of college GPA prediction models for first-time college students previously enrolled in animal sciences. During this five-year period, first-time college students enrolling in animal sciences at the land-grant institution achieved a first-semester GPA significantly lower than predicted. Because students consistently performed at a lower academic level than anticipated, these data suggest additional factors beyond standardized test score and high school GPA may need to be included in the model to improve prediction accuracy.

First-time college students enrolled in animal sciences in the fall of 2023 also underperformed their predicted GPA following their first semester. Interestingly, this changed after four semesters of coursework, and predicted GPA was no longer different than earned GPA. We conclude retained students achieved their predicted GPA after two years of coursework. The 13 students (11%) who were no longer at the university after four semesters had a significantly lower GPA than those retained. We conclude predicted GPA is an accurate academic success indicator for animal sciences undergraduate students. An implication of this conclusion is students with lower predicted GPAs are less likely to be retained at the university. Advisors should give special attention to students with lower predicted GPAs in terms of academic support, which may include changing majors.

The addition of critical thinking scores did not significantly improve the predictive GPA model for animal sciences first-time college students. Although critical thinking emerged as the predictor with the second highest standardized beta weight, the unique contribution was not significant to the model. We conclude the current predictive GPA model would not be significantly improved by the inclusion of the additional data. By using longitudinal GPA data, this study supports the current practice of using standardized test scores and high school GPA as consistent predictors of academic success of undergraduate animal sciences students.

Graduating class size and youth agricultural club involvement were neither practical nor significant predictors of student success as measured by GPA. These findings raise more questions than answers. Literature suggests student involvement in youth agriculture clubs facilitates responsibility and the development of critical thinking skills. Our data do not support school size or youth agricultural club involvement as independent data points which contributed to differences in predicted and earned GPA. We acknowledge we could not isolate individual contribution of these factors, which may exist, from current predictors. A dearth of literature has documented how specific youth agriculture experiences like showing animals, livestock judging, ownership projects, serving in leadership positions, etc. help build critical thinking skills. However, we recognize at least four limitations: (1) students have unique and varied experiences within these organizations, (2) asking a broad question about membership in youth agricultural organizations may fail to capture these unique experiences, (3) growth in critical thinking for students in agricultural youth organizations may be currently captured through existing prediction factors, and (4) student involvement in outside of school activities tends to be positively related to socio-economic status and may not represent all students. We must also acknowledge for these students, youth agricultural club membership may not impact critical thinking in ways measured by the CCTST or at all for this cohort. If we find the positive, we conclude the perceived limitations of being from a small school or not having agricultural youth club experience had little bearing on differences of predicted and earned GPA for these animal sciences students.

## Recommendations and discussion

The recommendations from this study include utilizing standardized tests, exploring new predictors for student academic achievement, better identifying students’ skills at enrollment and course registration, and encouraging continued development of critical thinking skills for current and future animal sciences undergraduate students.

Two recent changes in the use of standardized tests raise concerns. The first concern is the decision for ACT to remove the science portion of their test. The lack of a specific science score may make it more difficult to predict student success in STEM programs. We recommend further exploration into future cohorts to assess how this change may affect the current prediction model. A second trend to monitor is the growing popularity of “test-optional” admittance policies among many universities. Research published by ACT suggests their test assesses critical thinking skills (ACT 2025). Further, high school GPA, particularly in post-COVID education, has lost some of its predictive capacity for college success, as indicated by lower predicted GPA models (Sanchez 2024). Our data supports the validity of the current prediction model, which relies more heavily on high school core GPA, supplemented with standardized test data. We recommend animal sciences programs work with university administrators to explore the predicted and earned GPA of students, advising groups, and retention rates of students admitted test optional. To what extent are standardized test scores an important and unique component of GPA predictions for animal sciences undergraduates?

Interestingly, students’ involvement in youth agriculture clubs was not a unique contributor to exceeding their predicted animal sciences GPA. Many of these club experiences involve hands-on experience with animals. Today’s animal sciences students are relatively evenly split between rural and urban backgrounds, with less than half the students in any given year having livestock experience. When reflecting on the case, many students in the animal sciences program at this Midwestern land-grant institution initially express an interest in pursuing veterinary medicine and consistently show a desire to work with animals. However, a significant number of these students also express a lack of interest in chemistry and mathematics, which are key components of the animal sciences curriculum and require critical thinking (Carnevale et al. 2011). Assisting students in understanding the different aspects of their major and career choices could improve their chances of success and help them avoid feeling overwhelmed or out of place in their academic journey. In an effort to support all first-time students, regardless of credit standing, the study institution requires all first-time animal sciences students complete a one-semester Animal Sciences Orientation course during their first semester on campus. This course is designed to introduce students to the field of animal sciences, share the supportive resources available across campus, and describe additional experiential learning opportunities, including undergraduate research, internships, and study abroad programs. Student support and career readiness continues in a required Careers in Animal Sciences course, typically completed during the students’ second academic year. This course explores the array of careers within animal sciences and helps students to develop skills to obtain a career following graduation by creating a professional resume and cover letter, polishing interviewing skills, and assessing the value of a job offer. These courses aim to improve retention within the program and prepare students for success following completion of the program. However, further research should investigate the specific animal sciences hands-on experiences of animal sciences undergraduate students and to what extent those experiences are related to degree completion.

Students significantly underperformed their predicted GPA within the first semester, but those who were retained were able to meet their predicted GPA by semester four. This could be attributed to the advancement in the curriculum and enrollment in courses catered more toward the students’ interests. They have also had time to adjust to the rigors of college coursework and have perhaps established better studying and learning habits. We recommend further research into the internal and external factors into why and how first semester GPAs were lower, and why and how students were able to increase their GPAs. Although the current model is a significant predictor of earned GPA, questions remain regarding the unexplained 60% of the variance. The institution collects additional data on admitted students not used in the prediction model. These data could be explored to see if inclusion leads to a more accurate model. Literature points to factors like first-generation status, financial need, and personal traits like grit or perseverance (Strayhorn 2006; Lam and Zhou 2019) as important predictors of academic success.

When compared to previous cohorts, more current students have completed college credit courses during their high school education through dual credit or advanced placement options. This trend raises questions regarding changes in what is represented by high school core GPA and how students are advised when they reach the university. Dual credit courses often affect a student’s high school GPA as they are weighted more heavily. This can potentially inflate a student’s high school GPA, which could in turn increase their predicted GPA. The student’s success in a dual credit course may not accurately reflect their potential in higher education, often deceiving students to think they are ready for college (Zimmermann 2012). Students do not all have the same academic experiences prior to pursuing higher education, making it increasingly difficult to compare students with one another based on high-school assessments and grades (Allen 2005). Students who transfer a significant amount of college credit as first-time college students may be placed in upper-level courses because they have already completed many foundational or general education courses. This may inadvertently place students in courses for which they are not fully prepared. We recommend future research on how participation in dual credit is related to college GPA for animal sciences students. A recommendation for practice is for advisors to consider how dual-credit courses may weigh into predicted GPAs and consider academic readiness and student readiness when determining course schedules. We recommend further investigation into the dual credit coursework being transferred in by first-time animal sciences students and to what extent STEM coursework is related to grades in animal sciences courses.

Although previous students scored significantly lower than normative data on critical thinking and significantly below predicted GPA, the data do not support a causal relationship between critical thinking and academic success, beyond the critical thinking captured through the current predictive model (high school GPA and ACT). Critical thinking is a fundamental component of problem-solving across all levels of STEM (NRC 2006) and an in-demand trait of those in STEM disciplines (Oliver et al. 2010; Flores et al. 2012; Prinsley and Baranyai 2015). Critical thinking is essential not only in higher education and one’s professional life, but it is also applicable to everyday life (Halpern 2002; AACU 2010; Rogovaya et al. 2019). Why do critical thinking skills not further explain GPA? Are critical thinking skills a component of university GPA? If so, which ones? What is the nature of the relationship, if any, between critical thinking and the current predictive model (ACT and high school GPA)? Different majors require different skill sets for academic success. Is it possible to adjust predictive models specifically for STEM majors?

This finding leaves room to inquire why students with advanced critical thinking skills would not outperform their predicted GPA. Perhaps the underclassman animal sciences curriculum does not require critical thinking skills in their current format. A review of the program’s curriculum and course delivery could identify areas where critical thinking is required and being taught to better assist students in developing competencies relevant to the program and their professional development. Further research could explore the relationship between students’ current critical thinking scores and their standardized test scores or how many of the students that were above average achieved their predicted GPA. A follow-up study to compare students’ critical thinking scores to the normative data at the end of their animal sciences program could also be pursued in the coming years.

## List of abbreviations

IRB: Institutional Review Board
GPA: Grade Point Average
ACT: American College Testing
SAT: Scholastic Aptitude Test
SIS: Student Information System
CCTST: California Critical Thinking Skills Test
FFA: National FFA Organization
4-H: Extension-led youth development program
STEM: Science, Technology, Engineering, and Mathematics
VIF: Variance Inflation Factor
